# Supplementary material for: Psychiatric disorders comorbid with general medical illnesses and functional somatic disorders: The Lifelines cohort study
Source: PLoS One. 2023 May 30;18(5):e0286410. doi: 10.1371/journal.pone.0286410 (PMC10228816; doi:10.1371/journal.pone.0286410)
Supplement: S1 Table — (DOCX) [file pone.0286410.s001.docx]

**Table S1 Proportion of each diagnostic group with psychiatric disorder by sex**

|  | Female |  | Male |
| --- | --- | --- | --- |
| Inflammatory bowel disease | 90/707  12.7% |  | 24/389  6.2% |
| Irritable bowel syndrome | 1658/9301  17.8% |  | 263/2188  12.0% |
| Rheumatoid Arthritis | 209/1556 13.4% |  | 78/896 8.7% |
| Fibromyalgia | 669/3416  19/6% |  | 55/317  17.3% |
| Chronic Fatigue Syndrome | 272/989  27.5% |  | 107/417  26.6% |
| Diabetes | 192/1483  12.9% |  | 110/1278  8.6% |
