# Supplementary material for: Psychiatric disorders comorbid with general medical illnesses and functional somatic disorders: The Lifelines cohort study
Source: PLoS One. 2023 May 30;18(5):e0286410. doi: 10.1371/journal.pone.0286410 (PMC10228816; doi:10.1371/journal.pone.0286410)
Supplement: S2 Table — (DOCX) [file pone.0286410.s002.docx]

**Table S2 Participants with irritable bowel syndrome**

|  | No psychiatric disorder  N=9559 | One or more psychiatric disorder  N=1918 | P value |
| --- | --- | --- | --- |
| **Categorical variables** |  |  |  |
| %female | 79.9% | 86.3% | 0.001 |
| Few years education | 29.5% | 31.7% | <0.001 |
| Marr/cohabiting | 81.1 | 75.6 | <0.001 |
| Work f/t | 30.9% | 23.5% | <0.001 |
| Off sick | 4.1% | 10.4% | <0.001 |
| Low income | 16.3% | 23.7% | <0.001 |
| CFS | 3.0 | 6.4 | <0.001 |
| Fibromyalgia | 9.8 | 15.2 | <0.001 |
| IBD | 1.2 | 1.5 | ns |
| Life psych dis | 24.2% | 65.8% | <0.001 |
|  |  |  |  |
| **Continuous variables Mean (sd)** |  |  |  |
| Age | 43.9 (12.6) | 42.3 (11.6) | <0.001 |
| Life events and diffs score | 2.9 (1.7) | 3.7 (1.5) | <0.001 |
| No. of Gen med disorders | 1.8 (1.0) | 1.9 (1.0) | <0.001 |
| Chronic illness difficulties | 1.4 (0.6) | 1.7 (0.7) | <0.001 |
| Neuroticism | -4.8 (1.7) | -4.1 (1.9) | <0.001 |
| Social appreciation score | 24.8 (3.5) | 23.5 (3.9) | <0.001 |
| PSQI score | 4.5 (2.4) | 5.3 (3.0) | <0.001 |
| RAND items: |  |  |  |
| General health | 65.7 (13.8) | 59.5 (14.9) | <0.001 |
| Bodily pain | 74.8 (21.3) | 69.4 (23.9) | <0.001 |
| Physical functioning | 81.6 (17.1) | 70.9 (19.2) | <0.001 |
| Role physical | 79.3 (33.2) | 65.5 (40.3) | <0.001 |

PSQI =Pittsburgh Sleep Quality Inventory
