# Supplementary material for: Psychiatric disorders comorbid with general medical illnesses and functional somatic disorders: The Lifelines cohort study
Source: PLoS One. 2023 May 30;18(5):e0286410. doi: 10.1371/journal.pone.0286410 (PMC10228816; doi:10.1371/journal.pone.0286410)
Supplement: S3 Table — (DOCX) [file pone.0286410.s003.docx]

**Table S3 Participants with rheumatoid arthritis**

|  | No psych disorder  N=2165 | Psych disorder  N=287 | P value |  |
| --- | --- | --- | --- | --- |
| **Categorical variables** |  |  |  |  |
| %female | 62.2% | 72.8% | <0.001 |  |
| Few years education | 41.8 % | 47.9% | 0.002 |  |
| Marr/cohabiting | 84.0 | 75.6 | <0.001 |  |
| Work f/t | 28.6% | 19.5% | 0.001 |  |
| Off sick | 10.3% | 16.7% | <0.001 |  |
| Low income | 15.2% | 25.4% | <0.001 |  |
| smoked | 19.1 | 28.5% | <0.001 |  |
| IBS | 12.4% | 20.6% | <0.001 |  |
| CFS | 2.1 | 9.1 | <0.001 |  |
| Fibromyalgia | 9.5 | 19.9 | <0.001 |  |
| Life psych dis | 17.0% | 50.5% | <0.001 |  |
|  |  |  |  |  |
| **Continuous variables Mean (sd)** |  |  |  |  |
| Age | 52.7 (12.1) | 50.2 (11.2) | 0.001 |  |
| Life events and diffs score | 2.4 (1.7) | 3.4 (1.7) | <0.001 |  |
| No. of Gen med disorders | 2.8 (0.9) | 3.0 (1.7) | <0.001 |  |
| Chronic illness difficulties | 1.5 (0.6) | 1.8 (0.7) | <0.001 |  |
| Neuroticism | -5.0 (1.7) | -4.5 (1.8) | <0.001 |  |
| Social appreciation score | 25.1 (3.5) | 23.7 (3.9) | <0.001 |  |
| PSQI score | 4.4 (2.6) | 5.5 (3.3) | <0.001 |  |
| RAND items: |  |  |  |  |
| General health | 61.1 (14.7) | 55.4 (14.9) | <0.001 |  |
| Bodily pain | 71.3 (21.7) | 61.7 (25.8) | <0.001 |  |
| Physical functioning | 77.4 (20.9) | 71.9 (21.7)) | <0.001 |  |
| Role physical | 71.3 (39.7) | 55.2 (41.3) | <0.001 |  |
