# Supplementary material for: Psychiatric disorders comorbid with general medical illnesses and functional somatic disorders: The Lifelines cohort study
Source: PLoS One. 2023 May 30;18(5):e0286410. doi: 10.1371/journal.pone.0286410 (PMC10228816; doi:10.1371/journal.pone.0286410)
Supplement: S4 Table — (DOCX) [file pone.0286410.s004.docx]

**Table S4 Participants with fibromyalgia**

|  | No psych disorder  N=3009 | Psych disorder  N=724 | P value |  |
| --- | --- | --- | --- | --- |
| **Categorical variables** |  |  |  |  |
| % female | 91.3% | 92.4% | ns |  |
| Few years education | 42.4 % | 43.8% | ns |  |
| Marr/cohabiting | 83.8 | 77.5 | <0.001 |  |
| Work f/t | 17.5% | 13.7% | 0.012 |  |
| Off sick | 10.1% | 18.5% | <0.001 |  |
| Low income | 18.2% | 28.0% | <0.001 |  |
| smoked | 20.2 | 26.2 | Ns |  |
| IBS | 31.1% | 40.3% | <0.001 |  |
| CFS | 6.6% | 12.8% | <0.001 |  |
| Life psych dis | 27.3% | 65.3% | <0.001 |  |
|  |  |  |  |  |
| **Continuous variables**  **Mean (sd)** |  |  |  |  |
| Age | 49.1 (11.0) | 47.2 (10.3) | 0.001 |  |
| Life events and diffs score | 2.8 (1.7) | 3.7 (1.5) | <0.001 |  |
| No. of Gen med disorders | 2.0 (1.1) | 2.1 (1.2) | 0.019 |  |
| Chronic illness difficulties | 1.7 (0.7) | 2.0(0.7) | <0.001 |  |
| Neuroticism | -5.1 (1.7) | -4.4 (1.8) | <0.001 |  |
| Social appreciation score | 24.6 (3.6) | 22.9 (3.8) | <0.001 |  |
| PSQI score | 5.2 (2.8) | 5.9 (3.2) | <0.001 |  |
| RAND items: |  |  |  |  |
| General health | 59.7 (14.7) | 53.6 (14.3) | <0.001 |  |
| Bodily pain | 61.3 (20.9) | 53.7 (21.8) | <0.001 |  |
| Physical functioning | 72.7 (18.2) | 68.1 (21.7) | <0.001 |  |
| Role physical | 60.4 (40.7) | 42.6 (41.3) | <0.001 |  |
