# Supplementary material for: Psychiatric disorders comorbid with general medical illnesses and functional somatic disorders: The Lifelines cohort study
Source: PLoS One. 2023 May 30;18(5):e0286410. doi: 10.1371/journal.pone.0286410 (PMC10228816; doi:10.1371/journal.pone.0286410)
Supplement: S5 Table — (DOCX) [file pone.0286410.s005.docx]

**Table S5 Participants with chronic fatigue syndrome**

|  | No psych disorder  N=1027 | Psych disorder  N=379 | P value |  |
| --- | --- | --- | --- | --- |
| **Categorical variables** |  |  |  |  |
| % female | 69.8% | 71.8% | ns |  |
| Few years education | 32.2% | 41.1% | 0.003 |  |
| Marr/cohabiting | 72.4 | 68.0 | ns |  |
| Work f/t | 29.7% | 20.3% | <0.001 |  |
| Off sick | 15.8% | 24.3% | <0.001 |  |
| Low income | 25.9% | 33.1% | 0.008 |  |
| smoked | 21.9 | 28.6 | 0.011 |  |
| IBS | 27.7% | 32.2% | ns |  |
| CFS |  |  |  |  |
| Fibromyalgia | 19.4 | 24.5 | 0.034 |  |
| Life psych dis | 41.7% | 76.0% | <0.001 |  |
|  |  |  |  |  |
| **Continuous variables Mean (sd)** |  |  |  |  |
| Age | 45.1 (12.3) | 45.3 (11.0) | ns |  |
| Life events and diffs score | 3.3 (1.7) | 3.9 (1.5) | <0.001 |  |
| No. of Gen med disorders | 2.0 (1.1) | 2.1 (1.2) | ns |  |
| Chronic illness difficulties | 1.8 (0.7) | 2.1(0.7) | <0.001 |  |
| Neuroticism | -4.9 (1.8) | -3.9 (2.0) | <0.001 |  |
| Social appreciation score | 24.4 (3.8) | 22.8 (4.3) | <0.001 |  |
| PSQI score | 5.1 (2.8) | 6.1 (3.3) | <0.001 |  |
| RAND items: |  |  |  |  |
| General health | 57.2 (15.4) | 51.8 (14.1) | <0.001 |  |
| Bodily pain | 68.3 (23.7) | 62.1 (22.5) | <0.001 |  |
| Physical functioning | 76.9 (22.2) | 70.8 (25.1) | <0.001 |  |
| Role physical | 55.1 (41.7) | 46.8 (41.3) | <0.001 |  |
