# Supplementary material for: Psychiatric disorders comorbid with general medical illnesses and functional somatic disorders: The Lifelines cohort study
Source: PLoS One. 2023 May 30;18(5):e0286410. doi: 10.1371/journal.pone.0286410 (PMC10228816; doi:10.1371/journal.pone.0286410)
Supplement: S6 Table — (DOCX) [file pone.0286410.s006.docx]

**Table S6 Participants with diabetes**

|  | No psych disorder  N=2459 | Psych disorder  N=302 | P value |  |
| --- | --- | --- | --- | --- |
| **Categorical variables** |  |  |  |  |
| %female | 52.5% | 63.6% | <0.001 |  |
| Few years education | 47.2% | 50.5% | ns |  |
| Marr/cohabiting | 82.7 | 74.5 | 0.001 |  |
| Work f/t | 26.6% | 20.5% | 0.022 |  |
| Off sick | 7.2% | 17.2% | <0.001 |  |
| Low income | 16.6% | 25.3% | <0.001 |  |
| smoked | 14.7 | 25.1 | <0.001 |  |
| IBS | 9.8% | 20.2% | <0.001 |  |
| CFS | 1.6 | 7.0 | <0.001 |  |
| Fibromyalgia | 6.1 | 13.9 | <0.001 |  |
| Life psych dis | 6.1% | 13.9% | <0.001 |  |
|  |  |  |  |  |
| **Continuous variables Mean (sd)** |  |  |  |  |
| Age | 56.1 (12.1) | 51.8 (11.3) | ns |  |
| Life events and diffs score | 2.2 (1.7) | 3.3 (1.7) | <0.001 |  |
| No. of Gen med disorders | 1.7 (1.0) | 1.9 (1.1) | ns |  |
| Chronic illness difficulties | 1.5 (0.6) | 1.9 (0.7) | <0.001 |  |
| Neuroticism | -5.1 (1.6) | -4.4 (2.0) | <0.001 |  |
| Social appreciation score | 24.8 (3.8) | 23.6 (4.5) | <0.001 |  |
| PSQI score | 4.1 (2.4) | 5.2 (2.9) | <0.001 |  |
| RAND items: |  |  |  |  |
| General health | 63.1 (14.0) | 54.5 (14.6) | <0.001 |  |
| Bodily pain | 81.3 (21.3) | 68.7 (25.3) | <0.001 |  |
| Physical functioning | 84.5 (17.9) | 72.2 (25.8) | <0.001 |  |
| Role physical | 80.3 (31.2) | 61.7 (42.8) | <0.001 |  |
