# Supplementary material for: Psychiatric disorders comorbid with general medical illnesses and functional somatic disorders: The Lifelines cohort study
Source: PLoS One. 2023 May 30;18(5):e0286410. doi: 10.1371/journal.pone.0286410 (PMC10228816; doi:10.1371/journal.pone.0286410)
Supplement: Attachment — Submitted filename: comment.docx [file pone.0286410.s007.docx]

1. **Abstract:**

- Background is to short and it is good if definition of the outcome variable is included
- Line 25-26, “In a cross-sectional design logistic regression identified the variables most closely associated… “ better to rewrite it. Why cross-sectional design? Did you use it?
- Line 41, “**Abbreviations**” should be part of the declaration section of the manuscript.

2. **Introduction:**

- The magnitude of the problem should be described based on previously published literatures.
- You had better to include the gaps to which you want to fill and the aim of study in the introduction section of the manuscript.

3. **Methods and tools:**

- Who are your source population? Is
- What was the significance level of p value? It should be mentioned in the methods section.
- How valid and reliable your tools are?

4. **Result:**

- The continuous variables are presented as mean ± standard deviation (SD) in the tables. Were your data normally distributed? Have you checked the skewness? How much was it?

5. **Discussion:**

- Line 260-63, the statement “The variables closely associated with psychiatric disorder included those indicating predisposition to develop such disorders (neuroticism, and prior psychiatric disorder) as well as environmental variables including stressful life events and difficulties and chronic personal health difficulty.” Is incomplete and better to rewrite it.
- Line 267, avoid the statement *“*The strengths and limitations of this study must be recognized.”
- **Strengths and limitations** should be placed at the end of your discussion and just before conclusion.
- Poor justification of you findings. You have to justify more about your finding than justifying the others

6. **Conclusion:**

- What are your recommendations based on your current findings?

7. References

- Reference numbers 35, 36, 38, and 39 are relatively outdated and hence better to replace them with recent one if there is any.

8. You need to include your plan what did you do for those with severe problems if in case you find in your ethical approval? If you got such a study participant, it is unethical to disclose and leave them as they were.
9. You have to consider a major editorial issues of the whole document
10. The writing in English is very poor. There is poor use of punctuations and grammar in most parts of the text, I believe that an English language editor needs to revise the entire text before publication.
