# Supplementary material for: Psychiatric disorders comorbid with general medical illnesses and functional somatic disorders: The Lifelines cohort study
Source: PLoS One. 2023 May 30;18(5):e0286410. doi: 10.1371/journal.pone.0286410 (PMC10228816; doi:10.1371/journal.pone.0286410)
Supplement: Attachment — Submitted filename: Response to reviewers Creed May 2nd.docx [file pone.0286410.s009.docx]

Rebuttal Letter for PONE-D-22-33955R1 Psychiatric disorders comorbid with general medical illnesses and functional somatic disorders: The Lifelines cohort study

Thank you for the kind words regarding my previous revisions. Please find below the details of the further revisions I have made in the light of the reviewer’s additional points.

1. Introduction, p. 4, lines 87-92. Here, the author gives a justification for the hypothesis that different associations with psychiatric disorder exist in persons with mediccal conditions vs. those with functional disorders. I am sorry, but I did not understand the reasoning. Why the strongest correlation with joint pain in medical conditions and, among others, neuroticism in FSS? - This seems quite arbitrary. At least, some references supporting this argumentation seem to be necessary.

A new paragraph has been added to the introduction order to clarify the underlying purpose of this study (pages 3-4 lines 82-97):

The first analyses in this study examined whether psychiatric disorder was closely associated with a) the symptoms and impairment which accompany a medical or functional disorder, and/or b) a predisposition to develop psychiatric disorder, including neuroticism, psychosocial stress and prior psychiatric disorder which would be evident before the onset of the medical or functional disorder. Thus, for example, in rheumatoid arthritis it has been reported that the dominant predictors of self-reported depression are pain and fatigue and it is considered that these may be the main cause of the depression (41, 42). Depression in fibromyalgia is also associated with severity of pain and degree of impairment but, in addition, depression in fibromyalgia is closely associated with psychological variables such as neuroticism, learned helplessness, low self-efficacy and with psychosocial problems(43-46). These psychological variables are associated also with depression in the absence of physical illness (47-49). No previous study has assessed, across a range of disorders, whether depression comorbid with physical illness is associated both with the symptoms and impairment of the physical illness and with the psychological variables of neuroticism, prior psychiatric disorder, low social support and stressful life events unrelated to physical illness. In this study it was hypothesised that the correlates of psychiatric disorders would be similar in participants with functional somatic syndromes and general medical illnesses.

2. Introduction, p. 4, lines 95-99: Please support your statement on known predisposing and precipitating factors for psychiatric illness with authoritative references. This has been done (page 6, line 92). References 47-50 inserted:

47.	Bonsaksen T, Grimholt TK, Skogstad L, Lerdal A, Ekeberg Ø, Heir T, et al. Self-diagnosed depression in the Norwegian general population - associations with neuroticism, extraversion, optimism, and general self-efficacy. BMC public health. 2018;18(1):1076.

48.	Kendler KS, Gardner CO, Prescott CA. Toward a comprehensive developmental model for major depression in women. Am J Psychiatry. 2002;159(7):1133-45.

49.	Uliaszek AA, Zinbarg RE, Mineka S, Craske MG, Sutton JM, Griffith JW, et al. The role of neuroticism and extraversion in the stress-anxiety and stress-depression relationships. Anxiety Stress Coping. 2010;23(4):363-81.

50.	Kendler KS, Gardner CO, Prescott CA. Toward a comprehensive developmental model for major depression in men. Am J Psychiatry. 2006;163(1):115-24.

3. Methods, measures, p. 6, line 143-149/line 150-151: As far as I understood from the author's response, the list of 30 current of past medical illnesses also included the three functional disorders of interest. - If this is correct, this should be stated more clearly.

This has been made explicit (page 6, line 151-2):

“It included IBS, fibromyalgia and chronic fatigue syndrome.”

4. Methods, measures, p. 7, RAND instruments: The author responded to my comment on the standardization of the SF-36 items that all scores were computed and adjusted according to the recommended scoring system. However, table 1 stills shows values for four subscales of the SF-36 that do not adhere to the common metric (0-100). In fact, the mean of the subscale general health is negative ! - Thus, I cannot see that the standard procedure was applied to compute these four subscales of the SF-36. To my knowledge, the current scores cannot be compared to the multitude of studies that applied the SF-36, and the current scoring is not transparent. I'd like to suggest correcting the analyses using the SF-36 subscales.

Apologies. This has been done in tables 1, 3 and S2-S6.

5. Methods, measures: The author does not present data on the reliability of the measures. Information on Cronbach's alpha of all measures in the current sample would be helpful.

This requires access to raw data which are mostly unavailable to me now. I have been able to add the results for RAND items only – see page 8, Lines 187-8:

“Cronbach’s alpha for these items in this sample were: physical functioning = 0.87, Role functioning/ Physical = 0.89, Bodily Pain = 0.81, General Health = 0.80.”6. Methods, p. 8, statistical analysis, line 207: Typo - were the all the.

This has been corrected Page 9 line 214
